# The glucose infusion rate of parenteral nutrition in the first week of life in preterm infants: an observational study

**DOI:** 10.1186/s13052-021-01165-7

**Published:** 2021-11-04

**Authors:** Dina Angelika, Risa Etika, Martono Tri Utomo, Setya Mirha, Kartika Darma Handayani, I. Dewa Gede Ugrasena

**Affiliations:** 1 Faculty of Medicine, Universitas Airlangga – Dr. Soetomo General Academic Hospital, Surabaya, Indonesia; 2 Department of Child Health, Faculty of Medicine, Universitas Airlangga – Dr. Soetomo General Academic Hospital, Jl. Mayjend Prof. Dr. Moestopo No. 6-8, Airlangga, Gubeng, Surabaya, East Java 60286 Indonesia; 3 Department of Child Health, Faculty of Medicine, Universitas Brawijaya – Saiful Anwar General Hospital, Malang, Indonesia

**Keywords:** Parenteral nutrition, Glucose infusion rate, Morbidity, Mortality, Preterm infant

## Abstract

**Background:**

Most preterm infants require a continuous glucose infusion in the early postnatal period due to the interruption of the transplacental glucose supply after birth to promote better neurodevelopmental outcomes.

**Aims:**

To investigate the glucose infusion rate (GIR) on parenteral nutrition (PN) in the first week of life administered in preterm infants and its effect on neonatal morbidity and mortality.

**Methods:**

This study included 97 infants aged < 37 gestational weeks and weighed < 2500 g at birth. Infants recruited in this study were classified into 3 groups based on the GIR usage in parenteral nutrition as follows: GIR usage of 5- < 7 g/kg/day (Group I), GIR usage of 7–13 g/kg/day (Group II), and GIR usage of > 13–15 g/kg/day (Group III). Univariate and multivariate logistic regression analyzes were carried out to investigate whether the GIR usage in the three groups was associated with selected neonatal morbidities and mortality. Neonatal morbidities analyzed included respiratory distress syndrome (RDS), necrotizing enterocolitis, sepsis, retinopathy of prematurity, pulmonary hypertension, hypoglycemia, and hyperglycemia.

**Result:**

Of 97 preterm infants included, 51.5% infants had a gestational age of 34- < 37 weeks, and 54.6% infants had a birth weight of 1500- < 2500 g. The multivariate logistic regression analysis showed that the GIR usage of 5- < 7 g/kg/day was an independent variable that significantly increased the risk of hypoglycemia (Adjusted Odds Ratio [AOR] = 4.000, 95% Confidence Interval [CI] = 1.384–11.565, *P* = 0.010) and reduced the risk of sepsis (AOR = 0.096, 95% CI = 0.012–0.757, *P* = 0.026). The GIR usage in all three groups did not increase the risk of mortality. For neonatal morbidity analyzed in this study, RDS (AOR = 5.404, 95%CI = 1.421–20.548, *P* = 0.013) was an independent risk factor of mortality.

**Conclusion:**

The GIR usage of < 7 g/kg/day in PN in the first week of life administered to preterm infants was an independent variable in increasing hypoglycemia, but in contrast, reducing the risk of sepsis.

## Introduction

The early postnatal period is a critical phase for preterm infants due to interruption of transplacental nutrient transfer which requires adequate protein and energy intake from the beginning of birth to optimize long-term growth [1]. Parenteral nutrition (PN) is recommended for preterm infants who may not tolerate enteral feeding in the first few postnatal days, are critically ill and growing, have impaired bowel function due to diseases such as necrotizing enterocolitis, and have congenital anatomic gastrointestinal abnormalities [2, 3].. The significant improvements of PN in preterm infants developed in recent years resulted in more preterm infants survived and reduced poor neurodevelopmental outcomes [4]. The current provision of PN, referred to as early-aggressive PN, provides the administration of protein and energy at higher concentrations than the previous conventional PN, has been adopted as a standard service for neonatal care in many countries [5]. This PN practice must be administered directly after birth which aims to promote optimal plasma glucose levels and to ensure a positive energy balance [6].

As much as 60% of preterm infants require a continuous glucose infusion immediately after birth to maintain blood glucose levels [7, 8]. The glucose infusion rate (GIR) at parenteral nutrition in preterm infants should be maintained at 6–8 mg/kg/min to ascertain adequate glucose requirements [4, 7]. Some literature provides recommendations for glucose administration of PN in preterm infants with various GIRs in expressing carbohydrate intakes such as GIR of 8–12 g/kg/day [4], GIR of 6–15 g/kg/day [9], or GIR of 8–18 g/kg/day [10]. However, in early-aggressive PN, the GIR is enhanced to as high as 18 g/kg/day of glucose to attain optimal nutritional support which aims to improve growth and development outcome [9, 11]. A study conducted by Tottman et al. reported that decreasing carbohydrate intake of parenteral nutrition in preterm infants to 10.1 g/kg/day reduced the risk of neonatal hyperglycemia [12].

Either higher or lower glucose intake has a positive correlation with mortality [13, 14] and neonatal morbidity including respiratory distress syndrome (RDS) [15], necrotizing enterocolitis (NEC) [16], sepsis [17], retinopathy of prematurity (ROP) [18], pulmonary hypertension (PH) [19], hypoglycemia [20], and hyperglycemia [21]. Based on the description above, how much the glucose supply is optimal to provide a better outcome in preterm infants receiving parenteral nutrition is unclear and the results of related studies are conflicting [12, 13]. Therefore, this study aimed to investigate the GIR on PN in the first week of life administered in preterm infants and its effect on neonatal morbidity and mortality.

## Methods

### Study background and ethical approval

**Fig. 1:**
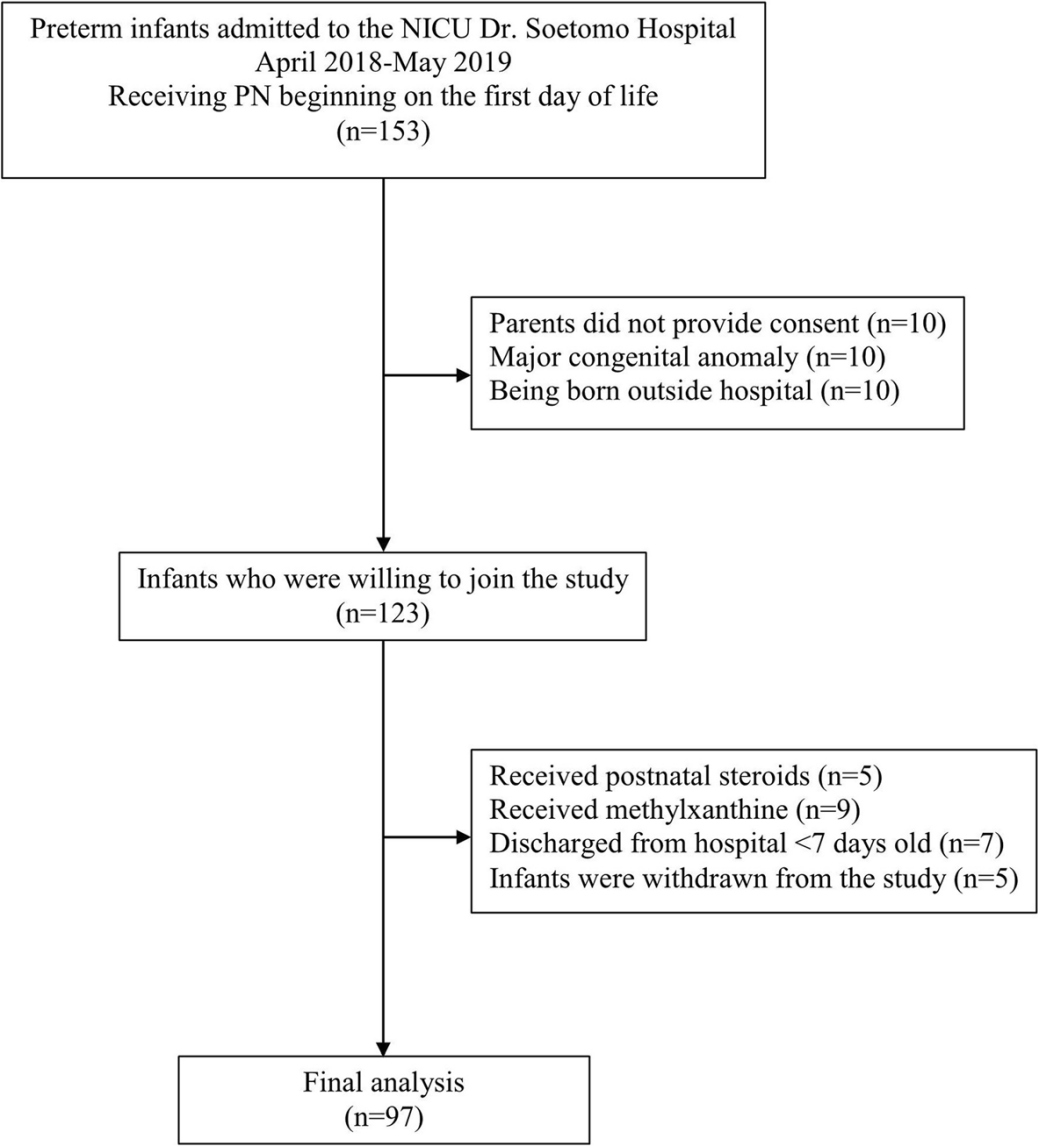
The study flow chart of participant recruitment

This observational cohort study was conducted in the neonatal intensive care unit (NICU) at Dr. Soetomo General Academic Hospital, Surabaya, Indonesia between April 2018 and May 2019. The sample size was calculated based on the formulation from Hulley et al. [22] with the following formula: an expected proportion of 0.5, a desired total width of 0.2, and a confidence interval (CI) of 95%; therefore, the calculation result was 97 samples. The study flow chart of subject recruitment was available in Fig. 1. Clinical data for each infant in this study were obtained through medical records.

This study was approved by the Ethical Committee in Health Research at the Dr. Soetomo General Academic Hospital (Reference number. 0203/KEPK/IV/2018). Informed and signed consent was acquired from the parents or authorized representatives of each infant.

### Participant eligibility

All preterm infants admitted to the NICU of the Dr. Soetomo General Hospital between April 2018 and May 2019 and receiving parenteral nutrition beginning on the first day of life were enrolled in this study. The exclusion criteria were: 1) being born outside this hospital, 2) receiving postnatal steroids and/or methylxanthine [15, 23], 3) having any major congenital anomaly [21], 4) being discharged from hospital in less than 7 days old, 5) the parents did not provide consent, or 6) infants were withdrawn from the study by their parents.

The risk of hypoglycemia due to prematurity or low birth weight and perinatal asphyxia could be considered as confounding factors that could influence the results of this study. Therefore, to control the risk of hypoglycemia due to prematurity or low birth weight, all samples in the study were preterm infants, in addition, the blood glucose (BG) levels analyzed were BG levels during PN. In controlling for asphyxia, the pediatric residents who were in charge of helping the infant during delivery were doctors trained in neonatal resuscitation.

Participants recruited in this study were classified into 3 groups based on the use of GIR in parenteral nutrition as follows: GIR of 5- < 7 g/kg/day (Group I), GIR of 7–13 g/kg/day (Group II), and GIR of > 13–15 g/kg/day (Group III) [9, 11]. The GIR value analyzed in this study was the highest GIR value given to each infant during the administration of parenteral nutrition in the first week of life. The study was conducted during the administration of parenteral nutrition in the first week of life.

### Nutrition protocol

All infants received PN according to the standard protocol in our NICU beginning on the first day of life. Intravenous amino acids (Aminosteril Infant; Fresenius Kabi, Bad Homburg, Germany) were started on the first day with a dose of 2 g/kg/day and increased daily by 0.5 g/kg/day to 3.5 g/kg/day [24].

Intravenous dextrose was administered with a GIR of at least 5 g/kg/day. If the infants could tolerate that dosage, on the next day, the GIR was increased by 1–2 g/kg/day adjusted based on daily fluid volume and BG level. The maximum GIR administered was 15 g/kg/day. The administration of a GIR exceeding 15 g/kg/day required careful consideration and discussion among supervisors. The BG level was maintained at a level of 45–150 mg/dL. PN was administered via central venous access by 4 Fr polyvinyl chloride umbilical catheter (Vygon, Ecouen, France) or by 1 Fr/28 G peripherally inserted central venous polyurethane catheter (Premicath; Vygon, Ecouen, France). When using peripheral venous access, the maximum concentration of dextrose administered is 12.5%.

Intravenous lipids (Smoflipid 20%; Fresenius Kabi, Bad Homburg, Germany) were administered starting with a dose of 1 g/kg/day and increased daily by 0.5 g/kg/day to 3 g/kg/day [25]. Several electrolytes were provided including sodium (2–4 mmol/kg/day), potassium (1–2 mmol/kg/day), magnesium (0.1–0.3 mmol/kg/day), calcium (0.6–1.5 mmol/kg/day), and phosphate (0.7 mmol/kg/day). Water-soluble vitamin (Soluvit® N; Fresenius Kabi, Bad Homburg, Germany) and fat-soluble vitamin (Vitalipid® N; Fresenius Kabi, Bad Homburg Germany) were added to the PN solution at doses of 1 mL/kg/day and 4 mL/kg/day, respectively [26]. The total volume of fluid started at 80 mL/kg/day, then increased daily by 10 mL/kg/day for the first 3 days, then by 20 mL/kg/day daily until the target volume reached 180 mL/kg/day.

Enteral nutrition was given on the first day of life at a dose of 10 mL/kg/day. If the infant tolerated this amount, the volume was increased by 20 mL/kg/day. The infants received breast milk or preterm formula milk if breast milk was not available. PN was stopped when the enteral volume had reached 120 mL/kg/day [27].

### Variables

Clinical variables were collected including sex, gestational age, birth weight, mortality, and selected neonatal morbidity including RDS, NEC, sepsis, ROP, PH, hypoglycemia, and hyperglycemia. RDS was characterized by the presence of signs of respiratory distress shortly after birth and confirmed by the presence of a typical radiological appearance on chest radiographs [28]. NEC was confirmed by the appearance of pneumatosis intestinalis, pneumoperitoneum, or portal venous gas on an abdominal radiograph [29]. Neonatal sepsis was defined as the presence of clinical manifestations of infection accompanied by positive blood cultures [30]. ROP was confirmed according to indirect ophthalmoscopy [18]. PH was confirmed using echocardiography [19]. Hypoglycemia was defined as the BG level was less than 45 mg/dL, whereas hyperglycemia was defined as the BG level was more than 150 mg/dL [20, 21].

### Data and statistical analysis

Quantitative data were described using mean, median, range, minimum, maximum, and standard deviation (SD). Qualitative data were described using frequency and percentage. Intergroup comparisons were analyzed using Chi-Square tests or Kruskal-Wallis tests. The univariate analysis was carried out to investigate whether the GIR usage in the three groups was associated with selected neonatal morbidities and mortality. The multivariate logistic regression analysis was conducted to analyze which was the independent variable of the three GIR groups (Group I, Group II, and Group II) for each of selected neonatal morbidities (RDS, NEC, sepsis, ROP, PH, hypoglycemia, hyperglycemia) and mortality. Statistical analyzes were also performed to investigate the association between selected neonatal morbidities and mortality.

Univariate analysis was performed using the chi-square test. Multivariate logistic regression analysis was performed using a backward stepwise logistic regression model. Variables with a *P*-value < 0.05 for adjusted odds ratio (AOR) of 95% confidence interval (CI) using multivariate logistic regression analysis were accepted as independent variables. All statistical analyses were performed using IBM SPSS Statistics 21 (IBM Corp., Armonk, NY, USA). A *P* value < 0.05 was considered to be statistically significant.

## Results

### Characteristics of participants

A total of 153 infants met the inclusion criteria and were included in this study. 10 parents did not provide consent, 10 infants were born outside this hospital, 5 infants received postnatal steroids, 9 infants received methylxanthine, 10 infants had a major congenital anomaly, 7 infants discharged from hospital < 7 days old, and 5 infants were withdrawn from the study by their parents; therefore the data of 97 infants were analyzed. Of the 97 infants, the majority, 50 (51.5%) infants had a gestational age of 34- < 37 weeks, and 53 (54.6%) infants had a birth weight of 1500- < 2500 g. The infants in this study had a minimum gestational age of 27 weeks, maximum gestational age of 36 weeks, with a mean of 33 weeks, and an SD of 2 weeks. The infants also had a minimum birth weight of 600 g, a maximum birth weight of 2400 g, a mean of 1564 g, and an SD of 440 g. As much as 74.2% of infants were delivered by cesarean section. Detailed characteristics of the infants recruited in this study were described in Table 1.

**Table 1:** Characteristics of Participants

Variables	n (%)
Gender	
Male	50 (51.5)
Female	47 (48.5)
Gestational age	
< 30 weeks	14 (14.4)
30 - < 34 weeks	33 (34.1)
34 - < 37 weeks	50 (51.5)
Birth weight	
< 1000 g	15 (15.5)
1000 - < 1500 g	29 (29.9)
1500 - < 2000 g	37 (38.1)
2000 - < 2500 g	16 (16.5)
Mode of delivery	
Spontaneous	25 (25.8)
Cesarean section	72 (74.2)
Antenatal steroid	
Yes	39 (40.2)
No	58 (59.8)
Outcome	
Mortality	17 (17.5)
Survive	80 (82.5)
The use of GIR	
Group I	21 (21.6)
Group II	67 (69.1)
Group III	9 (9.3)

Data were shown as number and percentage

GIR, glucose infusion rate; Group I, the GIR usage of 5- < 7 g/kg/day; Group II, the GIR usage of 7–13 g/kg/day; Group III, the GIR usage of > 13–15 g/kg/day

An analysis of infant characteristics based on the three groups as described in Table 2. There were no significant differences in sex, gestational age, birth weight, and mortality in the three groups. Meanwhile, in neonatal morbidity, there were significant differences in sepsis (*P* = 0.022) and hypoglycemia (*P* = 0.027) in the three groups.

**Table 2:** Characteristics of Infants Based on Glucose Infusion Rate

Variables	GIR			*p*
	Group I n (%) *n* = 21	Gorup II n (%) *n* = 67	Group III n (%) *n* = 9	
Gender				
Male	14 (66.7)	33 (49.3)	3 (33.3)	0.196
Female	7 (33.3)	34 (50.7)	6 (66.7)	
Gestational age				
< 30 weeks	0 (0.0)	12 (17.9)	2 (22.2)	0.179
30 - < 34 weeks	6 (28.6)	24 (35.8)	3 (33.3)	
34 - < 37 weeks	15 (71.4)	31 (46.3)	4 (44.5)	
Birth weight				
< 1000 g	1 (4.7)	12 (17.9)	2 (22.2)	0.151
1000 - < 1500 g	6 (28.7)	21 (31.3)	2 (22.2)	
1500 - < 2000 g	13 (61.9)	20 (29.9)	4 (44.5)	
2000 - < 2500 g	1 (4.7)	14 (20.9)	1 (11.1)	
Outcome				
Mortality	1 (4.7)	13 (19.4)	3 (33.3)	0.130
Survive	20 (95.3)	54 (80.6)	6 (66.7)	
Neonatal morbidity				
RDS	10 (47.6)	37 (55.2)	4 (44.5)	0.728
NEC	3 (14.3)	4 (5.9)	2 (22.2)	0.193
Sepsis	1 (4.7)	22 (32.8)	4 (44.5)	0.022*
ROP	3 (14.3)	7 (10.4)	0 (0.0)	0.498
Pulmonary hypertension	3 (14.3)	5 (7.5)	1 (11.1)	0.630
Hypoglycemia	9 (42.9)	11 (16.4)	1 (11.1)	0.027*
Hyperglycemia	2 (9.5)	9 (13.4)	2 (22.2)	0.645

Note: GIR, glucose infusion rate (mg/kg/min); RDS, respiratory distress syndrome; NEC, necrotizing enterocolitis; ROP, retinopathy of prematurity; *Significant *p* value < 0.05

### Distribution of the GIR values from day 1 to day 7

**Fig. 2:**
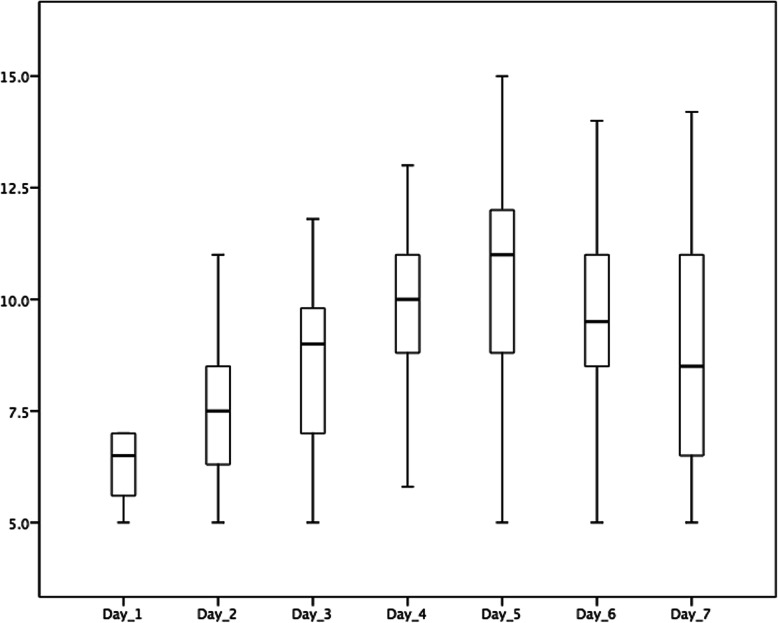
The distribution of glucose infusion rate (GIR) administered to parenteral nutrition in this study using the boxplot diagram. The X-axis described the time of the research which was conducted in days, started from day 1 to day 7. The Y-axis represented the level of the GIR which was defined in g/kg/day. The statistical test using the Kruskal-Wallis test showed that there was a significant difference in the GIR values in the three groups from day 1 to day 7 (*P* < 0.001)

The distribution of GIR values from day 1 to day 7 was described using the boxplot diagram in Fig. 2 as follows. The X-axis described the time of the research which was conducted in days, from the first day to the seventh day. The Y-axis represented the level of the glucose infusion rate which was defined in g/kg/day. Day 1, the median of GIR usage was 6.5 (range, 5–7) g/kg/day. Day 2, the median of GIR usage was 7.5 (range, 5–11) g/kg/day. Day 3, the median of GIR usage was 9 (range, 5–11.8) g/kg/day. Day 4, the median of GIR usage was 10 (range, 5–13) g/kg/day. Day 5, the median of GIR usage was 11 (range, 5–15) g/kg/day. Day 6, the median of GIR usage was 9.5 (range, 5–14) g/kg/day. Day 7, the median of GIR usage was 8.5 (range, 5–14.2) g/kg/day. The statistical test using the Kruskal-Wallis test showed that there was a significant difference in the GIR values in the three groups from day 1 to day 7 (*P* < 0.001).

### The association between the GIR usage in the three groups with neonatal morbidities

Univariate and multivariate logistic regression analysis was carried out to investigate whether the GIR usage in the three groups was associated with selected neonatal morbidities including RDS, NEC, sepsis, ROP, pulmonary hypertension, hypoglycemia, and hyperglycemia in each group (Table 3 and Table 4). The GIR usage in both group II and group III did not have a significant association with selected morbidity. Meanwhile, the statistical result showed that there was a significant association between the GIR usage in group I and hypoglycemia (odds ratio [OR] = 4.000, 95%CI = 1.384–11.565, *P* = 0.008). The multivariate logistic regression analysis in Table 4 showed that the GIR usage of 5- < 7 g/kg/day in a group I was a significant independent variable of increasing hypoglycemia risk (AOR = 4.000, 95% CI = 1.384–11.565, *P* = 0.010) and reducing sepsis risk (AOR = 0.096, 95% CI = 0.012–0.757, *P* = 0.026).

**Table 3:** Risk Factor of the three groups for selected Neonatal Morbidity

GIR	n (%)	OR	95% CI	*p*
Group I				
RDS	10 (47.6)	0.776	0.295–2.043	0.607
NEC	3 (14.3)	1.944	0.443–8.538	0.372
Sepsis	1 (4.7)	0.096	0.012–0.757	0.050
ROP	3 (14.3)	1.643	0.386–6.994	0.498
Pulmonary hypertension	3 (14.3)	1.944	0.443–8.538	0.372
Hypoglycemia	9 (42.9)	4.000	1.384–11.565	0.008*
Group II				
RDS	37 (55.2)	1.410	0.594–3.344	0.435
NEC	4 (5.9)	0.317	0.079–1.280	0.093
Sepsis	22 (32.8)	2.444	0.824–7.250	0.101
ROP	7 (10.4)	1.050	0.252–4.373	0.947
Pulmonary hypertension	5 (7.5)	0.524	0.130–2.109	0.257
Hypoglycemia	11 (16.4)	0.393	0.145–1.065	0.062
Hyperglycemia	9 (13.4)	1.009	0.285–3.575	0.989
Group III				
RDS	4 (44.5)	0.698	0.176–2.774	0.608
NEC	2 (22.2)	3.306	0.574–19.043	0.160
Sepsis	4 (44.5)	2.261	0.559–9.151	0.243
ROP	0 (0.0)	1.115	1.039–1.198	0.286
Pulmonary hypertension	1 (11.1)	1.250	0.138–11.307	0.842
Hyperglycemia	2 (22.2)	2.000	0.368–10.879	0.415

Note: GIR, glucose infusion rate (mg/kg/min); RDS, respiratory distress syndrome; NEC, necrotizing enterocolitis; ROP, retinopathy of prematurity; OR, odds ratio; CI, confidence interval; Group I, the GIR usage of 5- < 7 g/kg/day; Group II, the GIR usage of 7–13 g/kg/day; Group III, the GIR usage of > 13–15 g/kg/day

*Significant *p*-value < 0.05

**Table 4:** The result of multivariate logistic regression analysis

GIR	Neonatal Morbidity	AOR	95% CI for the AOR		P
Group I	Hypoglycemia, n (%)				
	9 (42.9)	4.000	1.384	11.565	0.010*
	Sepsis, n (%)				
	1 (4.8)	0.096	0.012	0.757	0.026*

GIR, glucose infusion rate; Group I, the GIR usage of 5- < 7 g/kg/day

AOR, adjusted odds ratio; CI, confidence interval

*A P value < 0.05 was accepted as the independent variable

### The association between the GIR usage in the three groups and neonatal morbidities with mortality

Univariate and multivariate logistic regression analysis was conducted to investigate the association between the GIR usage in the three groups and selected neonatal morbidities with mortality (Table 5 and Table 6). The statistical analysis showed that using a GIR of either GIR 5- < 7 g/kg/day, 7–13 g/kg/day, or > 13–15 g/kg/day did not increase the risk of mortality. In univariate analysis (Table 5), RDS (OR = 5.423, 95%CI = 1.446–20.346, *P* = 0.006) and sepsis (OR = 3.875, 95%CI = 1.306–11.495, *P* = 0.011) increased the risk of mortality. While in multivariate logistic regression analysis (Table 6), RDS was an independent risk factor of mortality (AOR = 5.404, 95%CI = 1.421–20.548, *P* = 0.013).

**Table 5:** Risk Factor for Mortality (*n* = 17)

Variables	n (%)	OR	95% CI	*p*
Group I	1 (5.9)	0.271	0.033–2.205	0.194
Group II	13 (76.5)	1.565	0.465–5.271	0.467
Group III	3 (17.6)	2.643	0.590–11.833	0.190
RDS	14 (82.4)	5.423	1.446–20.346	0.006*
NEC	2 (11.8)	1.390	0.263–7.363	0.691
Sepsis	9 (52.9)	3.875	1.306–11.495	0.011*
ROP	1 (5.9)	0.493	0.058–4.174	0.509
Pulmonary hypertension	3 (17.6)	2.643	0.590–11.833	0.190
Hypoglycemia	5 (29.4)	1.667	0.513–5.415	0.392
Hyperglycemia	2 (11.8)	0.836	0.168–4.171	0.827

Note: GIR, glucose infusion rate (mg/kg/min); Group I, the GIR usage of 5- < 7 g/kg/day; Group II, the GIR usage of 7–13 g/kg/day; Group III, the GIR usage of > 13–15 g/kg/day; RDS, respiratory distress syndrome; NEC, necrotizing enterocolitis; ROP, retinopathy of prematurity; OR, odds ratio; CI, confidence interval; *Significant *p*-value < 0.05

**Table 6:** The multivariate logistic regression analysis for mortality (*n* = 17)

Variable	Mortality n (%)	AOR	95% CI for AOR	*p*
Group I	1 (5.9)	0.189	1.023–1.563	0.122
RDS	14 (82.4)	5.404	1.421–20.548	0.013*

Note: Group I, the glucose infusion rate usage of 5- < 7 g/kg/day; RDS, respiratory distress syndrome; AOR, adjusted odds ratio; CI, confidence interval; *A *p*-value < 0.05 was accepted as independent variables

## Discussion

The significant improvement of nutritional strategies in preterm infants including early and aggressive parenteral nutrition resulted in more preterm infants survived and reduced detrimental outcomes [31]. Recently, the early-aggressive parenteral nutrition strategy has been adopted as a standard service for neonatal care in many countries [4, 13]. Since 2012, our NICU has also carried out an early-aggressive parenteral nutrition strategy as part of standard services in neonatal care. Amino acids and lipids were administered from the first day of life at doses of 2 g/kg/day and 1 g/kg/day, respectively. For glucose administration, the GIR provided was 5–15 g/kg per day which was adjusted based on daily fluid volume and BG level.

Glucose is the main source of non-protein calories in parenteral nutrition which is essential for the brain. The current parenteral nutrition management allows increasing the amount of glucose given to the infant while keeping the infant normoglycemic [6]. As much as 69.1% of preterm infants in this study received intravenous glucose with a GIR usage which was equivalent to a glucose supply of 7–13 g/kg/day. The amount of glucose administered in this study was consistent with a study conducted by Stensvold et al. which promoted the provision of early-aggressive parenteral nutrition which provided a glucose supply of 8.5 g/kg/day on the first day was then increased gradually to 15 g/kg/day [13]. Another study demonstrated a lower glucose administration to reduce the risk of hyperglycemia with a glucose supply of 10.1 g/kg/day in the first week [12]. However, some literature provides recommendations for administering glucose to PN with a wide variety of GIR such as GIR of 8–12 g/kg/day [4], or higher GIRs such as GIR of 6–15 g/kg/day [9] and GIR of 8–18 g/kg/day [10]. The most likely explanation for this wide variation in GIR is that the value of the maximum glucose oxidase capacity is not completely known in neonates. The rate of glucose administration for parenteral nutrition should exceed the maximum glucose oxidase capacity, which in neonates is possibly as high as 18 g/kg/day [11]. Furthermore, administration of the enteral route may support a lower GIR without risking hypoglycemia [12]. In this study, we did not quantify the amount of enteral nutrition; however, according to our protocol, we provided a minimum of enteral nutrition within 24 h after birth at a volume of 10 mL/kg/day, if tolerated then the enteral intake was increased by 20 mL/kg/d until reaching 180 mL/kg/day. PN was no longer administered when enteral nutrition reached a volume of 120 mL/kg/day.

Further analysis was carried out to investigate whether the GIR correlated with neonatal morbidity. This study found that the GIR usage of 5- < 7 g/kg/day significantly increased the risk of hypoglycemia. An initially delivered glucose intake at the hepatic glucose production of 6–8 mg/kg/min [32] or 6–10 g/kg/day [4] in early postnatal glucose infusion is necessary to prevent early postnatal hypoglycemia as a result of the interruption of the maternal-fetal glucose transfer and the low glycogen reserves of preterm infants. Another study demonstrated that to prevent hypoglycemia and meet the energy requirements needed for growth, it is recommended to start parenteral nutrition in preterm infants immediately after birth with a glucose intake at least equal to the basal glucose turnover rate for infants, 4–7 mg/kg/min [33]. Gluconeogenesis is the major pathway for glucose production in preterm infants; however, there is a delay in gluconeogenesis in the early days after the birth of preterm infants. Combined with low glycogen stores, this puts preterm infants at risk of hypoglycemia if they do not immediately receive exogenous glucose [34].

This study found that a GIR usage of 5- < 7 g/kg/day significantly reduced the risk of sepsis. These findings are consistent with a study by Tottman et al. which reported that a lower glucose administration reduced the risk of sepsis (10.1 g/kg/day versus 12.1 g/kg/day, *P* = 0.68) [12]. Other studies reported that administration of higher glucose increased the risk of sepsis [13, 31]. The explanation is that the subjects in that study were extremely low birth weight infants (gestational age of 30 weeks and birth weight of 1000 g), meanwhile, the majority of the infants recruited in this study were late-preterm infants with birth weights of 1500- < 2500 g. The late-preterm infants had a more mature digestive tract hence they did not receive PN for long, consequently such infants received less PN volume with a lower GIR. Based on our result, we suggest that further research is needed to further define glucose administration in parenteral nutrition in extremely low birth weight infants, particularly in a developing country.

The most common cause of hyperglycemia in the postnatal period is the high intake of glucose, in this case, the use of GIR > 10 mg/kg/min [35]. Early-aggressive parenteral nutrition, which allows an enhance in glucose supply, potentially increases the risk of hyperglycemia [13]. Based on our protocol, the highest limit of the GIR we use is 15 g/kg/day. In case the BG levels > 150 mg/dL, we reduced the rate of glucose infusion by 1–2 g/kg/day until BG levels return to normal levels; however, insulin is not a routine therapy in our NICU. We found that a GIR usage of > 13–15 g/kg/day did not increase the risk of hyperglycemia. We also found that using a GIR of either 7–13 g/kg/day or > 13–15 g/kg/day did not increase the risk of mortality. The occurrence of hyperglycemia at high GIR is partially due to increased catecholamine concentrations as a consequence of unstable and stressful conditions [36]. Current guidelines for PN practice in preterm infants recommended the early administration of amino acids and lipids. Evidence demonstrated that the introduction of amino acids within 4 h after birth diminished insulin-treated hyperglycemia incidence in preterm infants [37]. Our parenteral nutrition contains amino acids and lipids as macronutrients which we delivered within 24 h of birth to our preterm infants. The use of amino acids and lipids immediately after birth can improve glucose homeostasis, thereby reducing the risk of hyperglycemia.

This study found that RDS and sepsis significantly increased the risk of mortality, meanwhile, neither hyperglycemia nor hypoglycemia increased the risk of mortality. Based on the results of our study, hyperglycemia or hypoglycemia alone was not sufficient to affect mortality; however, mortality was related to the morbidity associated with both glucose imbalance [36].

This study had a few limitations that need to be considered. First, we did not quantify the amount of enteral nutrition. A previous study showed that the administration of enteral nutrition assisted glycaemic control [12]. Second, we did not perform urine glucose routinely. However, glycosuria is not a reliable biomarker for assessing blood glucose levels [38, 39]. Another limitation is that the group of preterm infants with birth weight < 1500 g or gestational age < 30 weeks as the main target of PN in preterm infants has a small portion in this study. Further studies are required to investigate what is the optimal GIR for such extremely low birth weight infants; however, the PN practice will continue to evolve.

## Conclusion

We found in this study that the GIR usage of < 7 g/kg/day in PN in the first week of life administered to preterm infants was an independent variable in increasing hypoglycemia, but in contrast, reducing the risk of sepsis. However, extremely low birth weight infants require further research.

## Data Availability

All data generated or analyzed during this study are included in this published article and its supplementary information files.
